# Trajectories of sickness absence and disability pension days among people with multiple sclerosis by type of occupation

**DOI:** 10.1177/13524585211048759

**Published:** 2021-10-06

**Authors:** Astrid R. Bosma, Chantelle Murley, Jenny Aspling, Jan Hillert, Frederieke G. Schaafsma, Johannes R. Anema, Cécile R.L. Boot, Kristina Alexanderson, Alejandra Machado, Emilie Friberg

**Affiliations:** Division of Insurance Medicine, Department of Clinical Neuroscience, Karolinska Institutet, SE-171 77 Stockholm, Sweden/ Department of Public and Occupational Health, Amsterdam Public Health Research Institute, Amsterdam UMC, VU University Amsterdam, Amsterdam, The Netherlands; Division of Insurance Medicine, Department of Clinical Neuroscience, Karolinska Institutet, SE-171 77 Stockholm, Sweden; Division of Insurance Medicine, Department of Clinical Neuroscience, Karolinska Institutet, SE-171 77 Stockholm, Sweden; Division of Neurology, Department of Clinical Neuroscience, Karolinska Institutet, SE-171 77 Stockholm, Sweden; Department of Public and Occupational Health, Amsterdam Public Health Research Institute, Amsterdam UMC, VU University Amsterdam, Amsterdam, The Netherlands; Department of Public and Occupational Health, Amsterdam Public Health Research Institute, Amsterdam UMC, VU University Amsterdam, Amsterdam, The Netherlands; Department of Public and Occupational Health, Amsterdam Public Health Research Institute, Amsterdam UMC, VU University Amsterdam, Amsterdam, The Netherlands; Division of Insurance Medicine, Department of Clinical Neuroscience, Karolinska Institutet, SE-171 77 Stockholm, Sweden; Division of Insurance Medicine, Department of Clinical Neuroscience, Karolinska Institutet, SE-171 77 Stockholm, Sweden; Division of Insurance Medicine, Department of Clinical Neuroscience, Karolinska Institutet, SE-171 77 Stockholm, Sweden

**Keywords:** Multiple sclerosis, occupation, sick leave, disability pension

## Abstract

**Background:**

Multiple sclerosis (MS) can impact working life, sickness absence (SA) and disability pension (DP). Different types of occupations involve different demands, which may be associated with trajectories of SA/DP among people with MS (PwMS).

**Objectives:**

To explore, among PwMS and references, if SA/DP differ according to type of occupation. Furthermore, to examine how trajectories of SA/DP days are associated with type of occupation among PwMS.

**Methods:**

A longitudinal nationwide Swedish register-based cohort study was conducted, including 6100 individuals with prevalent MS and 38,641 matched references from the population. Trajectories of SA/DP were identified with group-based trajectory modelling. Multinomial logistic regressions were estimated for associations between identified trajectories and occupations.

**Results:**

Increase of SA/DP over time was observed in all occupational groups, in both PwMS and references, with higher levels of SA/DP among PwMS. The lowest levels of SA/DP were observed among managers. Three trajectory groups of SA/DP were identified: Persistently Low (55.2%), Moderate Increasing (31.9%) and High Increasing (12.8%). Managers and those working in Science & Technology, and Economics, Social & Cultural were more likely to belong to the Persistently Low group.

**Conclusion:**

Results suggest that type of occupation plays a role in the level and course of SA/DP.

## Introduction

People are generally diagnosed with multiple sclerosis (MS) early in working life, with subsequent significant impact on work capacity and work participation. MS is a heterogeneous condition which can vary from mild symptoms to severe debilitating physical and/or cognitive limitations. MS symptoms, in combination with a progressive and unpredictable nature, may hinder people with MS (PwMS) to obtain or remain in paid work.^1,2^

Work participation rates among PwMS lag behind those of the general population, with PwMS more often being unemployed^2,3^ and holding higher levels of sickness absence (SA).^4,5^ In Sweden, a public insurance system is available in case of temporary or permanent reduced work capacity. Several demographic, disease-related and work-related factors are known to influence work capacity and work participation in PwMS.^6–8^ A mismatch between work demands and work capacity can result in a reduction in work-hours, changing job roles, or in SA or disability pension (DP).^9,10^ As physical and cognitive demands may vary between occupations, type of occupation might be associated with SA and DP.^11^ Less physical demanding jobs have been shown to increase time to DP and facilitate continued employment among PwMS.^12^

PwMS have previously been shown to have various patterns pre- and post-diagnosis of trajectories of SA and DP days. Although increasing and consistent high trajectories of SA/DP were identified, the majority of PwMS had a flat trajectory or a trajectory of marginally increasing SA/DP throughout the study period.^13^ It is reasonable to assume that type of occupation plays a role regarding SA and DP in PwMS; however, such associations remain relatively unexplored. Knowing whether PwMS in certain types of occupations pose higher risks of SA and DP could possibly facilitate interventions to promote extending working life. As far as our knowledge goes, no other study has explored the relationship between type of occupation (beyond white versus blue collar) and SA/DP over time among PwMS. The aim of this study was to explore, among prevalent PwMS (all individuals diagnosed with MS including both newly diagnosed and those with a longer disease duration) and references without MS, types of occupations and their respective annual levels of SA and DP. Furthermore, we aimed to gain more knowledge of how trajectories of SA and DP days are associated with type of occupation among PwMS.

## Methods

A population-based prospective cohort study including individuals with prevalent MS in 2010 and matched references without MS was conducted. Annual observations from 2010 through 2016 were included as follow-up years, with baseline characteristics at Y_−1_, representing 31 December 2009, as the year preceding follow-up.

Data from several Swedish nationwide registers, linked by unique personal identification number assigned to all residents in Sweden, were used. The Swedish MS Register (SMSReg) provides clinical data of individuals with MS in Sweden.^14^ The Longitudinal Integration Database for Health Insurance and Labour Market Studies (LISA) maintained by Statistics Sweden was used to obtain information on socio-demographic variables, type of occupation and for establishing the matched reference group. The Micro-Data for Analysis of the Social Insurance (MiDAS), maintained by the Swedish Social Insurance Agency, was used to retrieve annual SA and DP days. In Sweden, all workers – regardless of their occupation – have equal rights to the social insurances SA and DP. This insurance is not an employee benefit in the contract negotiation, as in other countries, but a legal entitlement to these social insurances for all residents in Sweden. All residents from 16 years of age with minimum income from work, unemployment benefits and other sources such as parental leave and student allowances can apply for SA if not being able to work due to injury or morbidity.Similarly, all aged 19–64, irrespective of previous income, can apply for DP if long-term or permanently unable to work due to morbidity. Furthermore, SA and DP days are granted either full-time (100%) or part-time (25, 50 or 75%) and can be granted in combination. Annual SA or DP net days (i.e. 2 days of 50% SA or DP equals 1 SA or DP net day) were combined and used for constructing the SA/DP outcome variable. Year of death was extracted from the Cause of Death Register, maintained by the National Board of Health and Welfare. Moreover, the National In- and Out-Patient Register (NPR) from the National Board of Health and Welfare was used to ensure the absence of MS diagnoses among possible references. From the same authority, the Swedish Prescribed Drug Register (SPDR) and the Cancer Register were used for construction of the Comorbidity Index.

We identified 8197 working-aged PwMS (19–57 years, that is, being of working-age during the whole follow-up) diagnosed with MS when 18 years or older, in or before 2010 (Supplementary Figure 1). Of those, 874 had missing year of diagnosis but had a year of onset listed and were included if the year of onset was in 2010 or earlier. As shown in Supplementary Figure 1, the following were not included: PwMS missing both year of diagnosis and year of onset, and those with paediatric onset MS – the latter as they are considered to have a different course and prognosis than those with adult-onset MS.^15^ A comparable reference group from the general population (*n* = 40,985), without MS diagnosis, was created by matching on sex, age, type of living area and county. For each MS individual, up to five references were included (see Supplementary Table 1 for baseline characteristics of both cohorts).

To assure that PwMS were available for work at the start of follow-up, individuals who already had DP at an extent over 50% in 2009 (i.e. more than 183 net DP days) were excluded, resulting in a final sample of 6100 individuals with MS and 38,641 references. Individuals were followed until year of death, emigration or end of 2016, whichever occurred first. At the end of follow-up (2016), 98% of cases and 97% of references remained.

The Swedish Standard of Classification of Occupations 1996 (SSYK) was used to classify jobs into the following occupational groups: (1) Managers across all sectors, (2) Science & Technology, (3) Healthcare, (4) Economics, Social & Cultural, (5) Education, (6) Administration, (7) Sales, (8) Construction and (9) Other (representing unspecified and other occupations, like military and agriculture) (see Supplementary Table 2 for more details on this occupational categorisation).

In this study, SA and DP net days were combined into one outcome variable (SA/DP), as the sum of mean annual SA/DP net days with a maximum of 366 days/year.

Socio-demographic characteristics were obtained for 2009 (Y_−1_) and included: sex, age, educational level, country of birth, county, type of living area (based on population density), marital status, and if living with children under 18 years of age. Moreover, information on employment status and payments from student allowances were gathered. In addition, comorbidity was assessed by constructing a modified Rx-Risk Comorbidity Index,^16,17^ by using the SPDR and the Swedish Cancer Register, where MS medication was excluded. For PwMS, disease duration was calculated from the first year of follow-up (2010) and year of MS diagnosis. Two categories to describe type of MS at diagnosis^18^ were included: (1) relapsing-remitting (RR) (also including PwMS with secondary progressive MS) and (2) primary progressive (PP) (also including primary remitting, as well as missing type of MS at diagnosis (3%) – only for descriptive purposes). The reasoning behind this classification was based on the presumption that several RR patients will convert to SP. Moreover, this classification differentiates between a more aggressive type of disease initiation (PP) and those diagnosed as RR.

Descriptive statistics were used for socio-demographic variables and types of occupations. Mean annual SA/DP net days during follow-up years were calculated and stratified by sex, disease duration and type of occupation.

Trajectories of mean annual SA/SP net days were estimated for PwMS during follow-up using group-based trajectory modelling (GBTM).^19^ The GBTM method identifies groups of individuals (trajectory groups) which follow a distinct pattern of SA/DP over time.^19^ Regression models were estimated for each trajectory group. Bayesian information criterion (BIC) and group belonging probability were used to determine the best-fitting model. Subsequently, PwMS were assigned to trajectories with highest probability of belonging. Associations were assessed with multinomial logistic regression. Crude and mutually adjusted (for socio-demographic, MS-related and occupational variables) analysis were performed. Odds ratios (ORs) and 95% confidence intervals (CIs) were calculated.

This project was approved by the Regional Ethical Review Board in Stockholm, Sweden.

## Results

Based on the DP exclusion criteria (i.e. more than 183 DP net days in 2009), relatively more PwMS, and PwMS of higher age, were excluded compared with references. Among the 6100 PwMS remaining, the average age was 40.9 (SD = 9.2), being predominantly women (71.4%), compared with an average age of 42.3 (SD = 9.2) and 71.7% women among references (Table 1). In 2010, PwMS had an average disease duration of 7.2 years and the prevailing type was relapsing-remitting MS (92.4%).

**Table 1.** Baseline characteristics in 2009 of people with MS (PwMS) and references.

	PwMS *n* = 6100 (%)	References *n* = 38,641 (%)
Sex		
Women	4355 (71.4)	27,689 (71.7)
Men	1745 (28.6)	10,952 (28.3)
Age (years)		
19–24	257 (4.2)	1295 (3.3)
25–34	1319 (21.6)	7054 (18.2)
35–44	2198 (36.0)	13,119 (34.0)
45–54	1895 (31.1)	13,593 (35.2)
55–57	431 (7.1)	3580 (9.3)
Mean age (SD)	40.9 (9.2)	42.3 (9.2)
Educational level		
Low: Compulsory school ⩽ 9 years^a^	465 (7.6)	4463 (11.5)
Medium: Upper secondary school 10–12 years	2794 (45.8)	17,995 (46.6)
High: Higher education > 12 years	2841 (46.6)	16,183 (41.9)
Married or in civil partnership		
No	3358 (55.0)	20,146 (52.1)
Yes	2742 (45.0)	18,495 (47.9)
Living with children aged <18 years		
No	3230 (53.0)	19,326 (50.0)
Yes	2870 (47.0)	19,315 (50.0)
Country of birth		
Sweden	5524 (90.6)	31,645 (81.9)
Outside of Sweden	576 (9.4)	6996 (18.1)
Type of living area		
Larger cities	2521 (41.3)	15,448 (40.0)
Medium-sized municipalities	2090 (34.3)	13,308 (34.4)
Smaller municipalities	1489 (24.4)	9885 (25.6)
Type of occupation		
Managers across all sectors	216 (3.5)	1797 (4.6)
Science & Technology	526 (8.6)	2652 (6.9)
Healthcare	1177 (19.3)	8316 (21.5)
Economics, Social & Cultural	797 (13.1)	3929 (10.2)
Education	458 (7.5)	3068 (7.9)
Administration	1108 (18.2)	5674 (14.7)
Sales	360 (5.9)	2075 (5.4)
Construction	904 (14.8)	6349 (16.4)
Other	554 (9.1)	4781 (12.4)
Employment status^b^		
In paid work	5229 (85.7)	32,739 (84.7)
Not in paid work	871 (14.3)	5902 (15.3)
Receiving parental leave benefits		
No	5149 (84.4)	32680 (84.6)
Yes	951 (15.6)	5961 (15.4)
Receiving student allowances		
No	5763 (94.5)	36,474 (94.4)
Yes	337 (5.5)	2167 (5.6)
SA/DP net days		
Mean SA/DP net days 2009	65	13
Comorbidity (categories)^c^		
0	772 (12.7)	12 784 (33.1)
1–2	3350 (54.9)	18 566 (48.0)
3–4	1439 (23.6)	5415 (14.0)
⩾5	539 (8.8)	1876 (4.9)
Disease duration		
0–4 years	2537 (41.6)	–
5–9 years	1820 (29.8)	–
10–19 years	1412 (23.2)	–
⩾20 years	331 (5.4)	–
Mean disease duration in years (SD)	7.2 (6.3)	–
Type of MS		
Relapsing-remitting	5635 (92.4)	–
Primary progressive	465 (7.6)	–

MS: multiple sclerosis; PwMS: people with MS; SD: standard deviation; SA: sickness absence; DP: disability pension; SPDR: Swedish Prescribed Drug Register.

Individuals with >50% on disability pension (DP) at baseline were excluded from the final sample. Variables are described by frequencies and percentages or mean and standard deviation (SD). MS-related variables were described solely for all PwMS.

a Individuals with missing variables added to lowest category, <0.75% of the cohort.

b Individuals unemployed, on parental leave or students (receiving student allowances) were included as ‘not in paid work’.

c Comorbidities are based on the SPDR and Swedish Cancer Register and categorized by a number of distinct comorbidity groups. The total number of comorbidities excludes MS.

The course of SA and DP days at follow-up showed a steady increase in mean annual SA/DP net days among both PwMS and reference group, with women having higher numbers of SA/DP net days than men (Supplementary Figure 2a). Moreover, mean SA/DP net days/year were higher among PwMS with longer disease duration (Supplementary Figure 2b). Complementary analysis of mean annual SA/DP net days/year according to type of MS is found in Supplementary Table 3.

**Figure 1.**
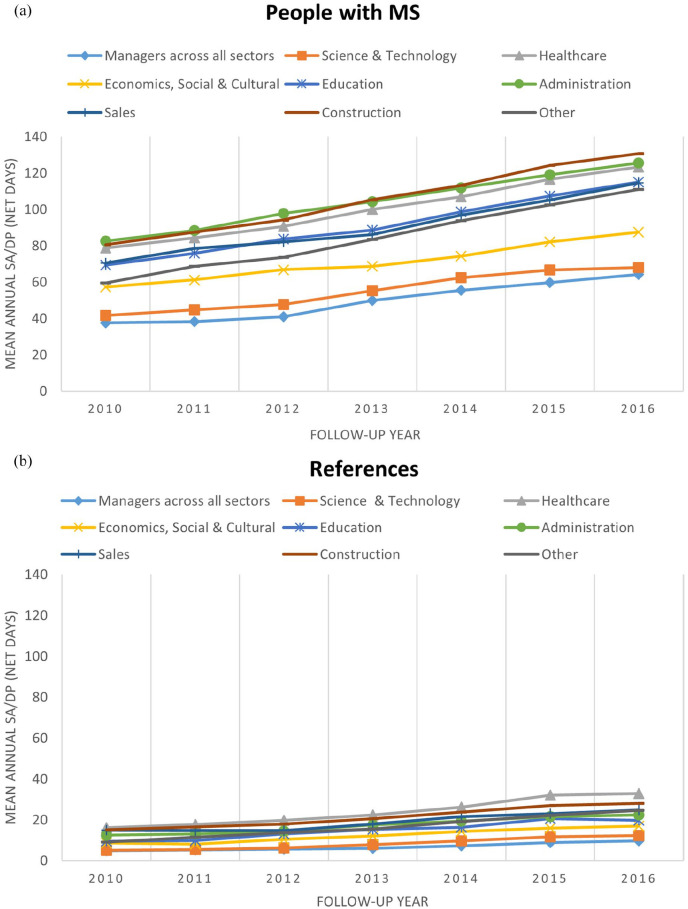
(a) Mean annual sickness absence (SA)/disability pension (DP) for people with MS (PwMS) and (b) references stratified by type of occupation during the 6-year follow-up.

**Figure 2.**
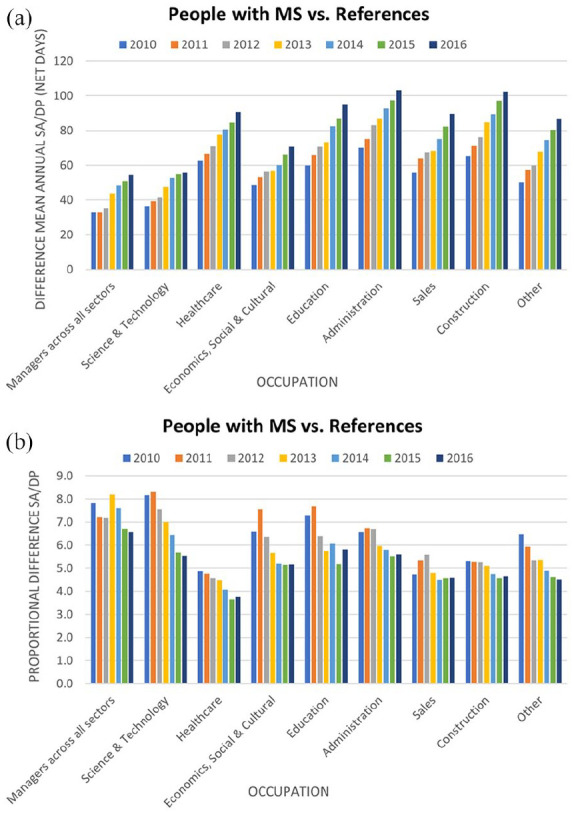
Difference in mean annual sickness absence (SA)/disability pension (DP) (a) and the proportional difference (b) between people with MS (PwMS) and references stratified by type of occupation. More variation over follow-up and per type of occupation was seen in (b), with some occupations showing a steady decrease (e.g. Healthcare), while others (e.g. Sales) showed an increase in the earlier years of follow-up, followed by decreasing proportional difference of mean annual SA/DP net days. The difference in mean annual SA/DP (a) was calculated as the subtraction of the average SA/DP net days between groups whereas the proportional difference (b) represents the ratio of SA/DP net days of PwMS and SA/DP net days of references – in both cases, these SA/DP measures show higher rates among PwMS when compared to references.

Regarding the type of occupations, among PwMS, Managers across all sectors had the lowest SA/DP net days, while Administration and Construction had the highest SA/DP net days. Comparable to PwMS, Managers among references also had the lowest SA/DP net days; however, Healthcare workers presented the highest SA/DP net days (Figure 1(a) and (b), respectively). The absolute difference of SA/DP net days between PwMS and references varied across types of occupations, with greater differences in Administration, Healthcare and Construction. However, when calculating the proportional difference (i.e. the ratio of SA/DP net days of PwMS and SA/DP net days of references), Managers among PwMS had about a sevenfold higher level of SA/DP net days than Managers among references, whereas for Healthcare sector, the corresponding difference was fourfold (Figure 2(a) and (b), respectively). Further information on full DP dropout within the follow-up years among PwMS and references can be found in Supplementary Table 4.

An overview of types of occupations and a summary of PwMS in these occupational groups is provided in Table 2. Managers had the highest mean age (44.7) compared with Sales (37.6) and Other (37.3) occupations. Education was the type of occupation with the greatest percentage of higher educated PwMS (93.7%) compared with Sales (22.2%). PwMS with longest disease duration were in Economics, Social & Cultural (8.1 years) and Administration (8.0 years), while the shortest average disease duration was observed among the occupations Sales (6.1 years), Construction (6.1 years) and Other (6.0 years).

**Table 2.** Baseline characteristics of people with MS (PwMS) by type of occupation.

	Managers across all sectors *n* = 216 (%)	Science & Technology *n* = 526 (%)	Healthcare *n* = 1177 (%)	Economics, Social & Cultural *n* = 797 (%)	Education *n* = 458 (%)	Administration *n* = 1108 (%)	Sales *n* = 360 (%)	Construction *n* = 904 (%)	Other *n* = 554 (%)
Sex									
Women	124 (57.4)	235 (44.7)	1090 (92.6)	648 (81.3)	395 (86.2)	884 (79.8)	271 (75.3)	356 (39.4)	352 (63.5)
Men	92 (42.6)	291 (55.3)	87 (7.4)	149 (18.7)	63 (13.8)	224 (20.2)	89 (24.7)	548 (60.6)	202 (36.5)
Age (years)									
19–24	0 (0)	4 (0.8)	38 (3.2)	7 (0.9)	2 (0.4)	25 (2.3)	46 (12.8)	54 (6.0)	81 (14.7)
25–34	19 (8.8)	117 (22.2)	239 (20.3)	142 (17.8)	107 (23.4)	238 (21.5)	96 (26.7)	212 (23.5)	149 (26.9)
35–44	86 (39.8)	252 (47.9)	390 (33.1)	298 (37.4)	161 (35.1)	395 (35.6)	126 (35.0)	333 (36.8)	157 (28.4)
45–54	85 (39.4)	123 (23.4)	409 (34.8)	279 (35.0)	146 (31.9)	365 (32.9)	79 (21.9)	265 (29.3)	144 (26.0)
55–57	26 (12.0)	30 (5.7)	101 (8.6)	71 (8.9)	42 (9.2)	85 (7.7)	13 (3.6)	40 (4.4)	23 (4.0)
Mean age (SD)	44.7 (7.5)	40.7 (7.9)	41.8 (9.2)	42.7 (8.5)	42.0 (9.1)	41.8 (8.9)	37.6 (9.6)	39.8 (9.1)	37.3 (10.5)
Country of birth									
Sweden	206 (95.4)	495 (94.1)	1069 (90.8)	747 (93.7)	424 (92.6)	1026 (92.6)	341 (94.7)	804 (88.9)	412 (74.3)
Outside of Sweden	10 (4.6)	31 (5.9)	108 (9.2)	50 (6.3)	34 (7.4)	82 (7.4)	19 (5.3)	100 (11.1)	142 (25.7)
Educational level									
Low: Compulsory school ⩽ 9 years^a^	9 (4.2)	11 (2.1)	40 (3.4)	12 (1.5)	5 (1.1)	87 (7.8)	65 (18.1)	146 (16.2)	90 (16.3)
Medium: Upper secondary school 10–12 years	59 (27.3)	131 (24.9)	575 (48.9)	219 (27.5)	24 (5.2)	672 (60.7)	215 (59.7)	654 (72.3)	245 (44.3)
High: Higher education > 12 years	148 (68.5)	384 (73.0)	562 (47.7)	566 (71.0)	429 (93.7)	349 (31.5)	80 (22.2)	104 (11.5)	219 (39.4)
Disease duration									
0–4 years	86 (39.8)	201 (38.2)	480 (40.8)	293 (36.8)	174 (38.0)	397 (35.8)	160 (44.5)	464 (51.3)	283 (51.0)
5–9 years	71 (32.9)	173 (32.9)	353 (30.0)	230 (28.8)	144 (31.4)	342 (30.9)	125 (34.7)	230 (25.4)	151 (27.3)
10–19 years	51 (23.6)	132 (25.1)	272 (23.1)	215 (27.0)	116 (25.3)	294 (26.5)	68 (18,9)	165 (18.3)	99 (17.9)
⩾20 years	8 (3.7)	20 (3.8)	72 (6.1)	59 (7.4)	24 (5.3)	75 (6.8)	7 (1.9)	45 (5.0)	21 (3.8)
Mean disease duration in years (SD)	7.2 (5.6)	7.2 (5.8)	7.4 (6.6)	8.1 (6.5)	7.5 (6.5)	8.0 (6.6)	6.1 (5.1)	6.1 (6.1)	6.0 (6.0)
Type of MS									
Relapsing-remitting	199 (92.1)	490 (93.2)	1114 (94.6)	731 (91.7)	427 (93.2)	1011 (91.2)	339 (94.2)	815 (90.2)	509 (91.9)
Primary progressive	17 (7.9)	36 (6.8)	63 (5.4)	66 (8.3)	31 (6.8)	97 (8.8)	21 (5.8)	89 (9.8)	45 (8.1)

MS: multiple sclerosis; PwMS: people with MS; SD: standard deviation.

Variables are described by frequencies and percentages or mean and standard deviation (SD).

a Individuals with missing variables added to lowest category, <0.75% of the cohort.

**Figure 3.**
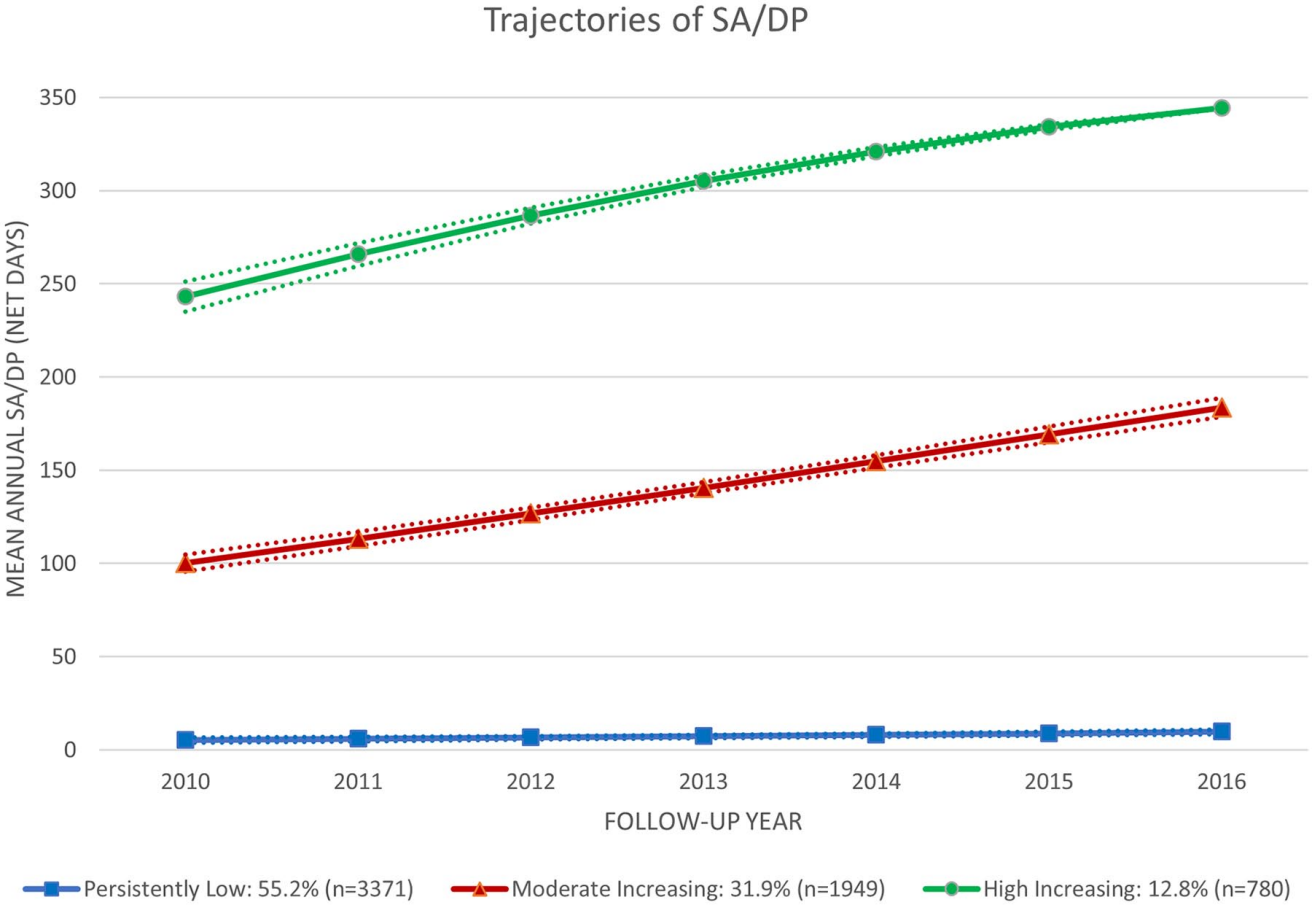
Trajectories of mean annual sickness absence (SA)/disability pension (DP) (net days) among people with MS. Baseline characteristics of these trajectory groups can be found in Table 5 of the supplementary files.

Three trajectory groups regarding mean SA/DP days/year among PwMS were identified (Figure 3) and named *Persistently Low* (group 1), *Moderate Increasing* (group 2) and *High Increasing* (group 3). Approximately, half of the PwMS (55.2%) belonged to the *Persistently Low*, followed by the *Moderate Increasing* (31.9%), and *High Increasing* (12.8%) group, respectively (for baseline characteristics of these trajectory groups, please see Supplementary Table 5).

Crude and adjusted associations of the *Moderate Increasing* and *High Increasing* groups compared with the *Persistently Low* group are shown in Table 3. In the adjusted model, low educational level (OR = 2.07, 95% CI = 1.64–2.60) and disease duration of ⩾20 years (OR = 1.57, 95% CI = 1.20–2.04) were associated with belonging to the *Moderate Increasing* group. Age range of 45–54 years (OR = 2.69, 95% CI = 2.08–3.46) was associated with the *High Increasing* group. Having ⩾5 comorbidities was strongly associated with both the *Moderate Increasing* (OR = 5.42, 95% CI = 4.18–7.04) and *High Increasing* (OR = 4.19, 95% CI = 3.00–5.85) groups.

**Table 3.** Factors associated with the sickness absence (SA)/disability pension (DP) trajectory groups among people with MS, using the persistently low group (group 1) as the reference group (*n* = 3371).

	Crude moderate increasing *n* = 1949	Crude high increasing *n* = 780	Adjusted moderate increasing *n* = 1949	Adjusted high increasing *n* = 780
Crude and adjusted^a^ odds ratio (95% confidence interval)				
Sex				
Women	**1.61 (1.44–1.81)**	1.00 (0.85–1.18)	**1.57 (1.37–1.80)**	1.04 (0.86–1.27)
Men	Reference	Reference	Reference	Reference
Age (years)				
19–24	**0.53 (0.38–0.74)**	**0.36 (0.17–0.79)**	**0.53 (0.37–0.76)**	**0.30 (0.14–0.65)**
25–34	Reference	Reference	Reference	Reference
35–44	**1.62 (1.40–1.87)**	**1.61 (1.26–2.06)**	**1.51 (1.29–1.76)**	**1.63 (1.26–2.10)**
45–54	**3.05 (2.63–3.54)**	**2.97 (2.34–3.77)**	**2.45 (2.08–2.89)**	**2.69 (2.08–3.46)**
55–57	**3.52 (2.81–4.41)**	**2.89 (2.10–3.99)**	**2.75 (2.14–3.54)**	**2.55 (1.80–3.60)**
Educational level				
Low: Compulsory school ⩽ 9 years^b^	**2.10 (1.72–2.56)**	**2.71 (2.10–3.50)**	**2.07 (1.64–2.60)**	**1.95 (1.45–2.61)**
Medium: Upper secondary school 10–12 years	**1.57 (1.41–1.75)**	**1.79 (1.51–2.11)**	**1.45 (1.27–1.65)**	**1.45 (1.20–1.76)**
High: Higher education > 12 years	Reference	Reference	Reference	Reference
Country of birth				
Sweden	Reference	Reference	Reference	Reference
Outside of Sweden	0.89 (0.75–1.06)	1.26 (0.99–1.60)	1.02 (0.84–1.24)	**1.36 (1.05–1.77)**
Type of living area				
Larger cities	Reference	Reference	Reference	Reference
Medium-sized municipalities	**1.36 (1.21–1.53)**	**1.33 (1.11–1.59)**	**1.29 (1.13–1.47)**	**1.27 (1.05–1.53)**
Smaller municipalities	**1.74 (1.53–1.98)**	**1.51 (1.25–1.83)**	**1.44 (1.25–1.67)**	**1.25 (1.02–1.53)**
Type of occupation				
Managers across all sectors	**0.33 (0.24–0.46)**	**0.52 (0.31–0.86)**	**0.37 (0.26–0.52)**	**0.52 (0.30–0.89)**
Science & Technology	**0.40 (0.32–0.50)**	**0.39 (0.27–0.58)**	**0.64 (0.50–0.82)**	**0.58 (0.39–0.88)**
Healthcare	0.99 (0.84–1.17)	0.89 (0.71–1.13)	0.93 (0.78–1.11)	0.89 (0.69–1.14)
Economics, Social & Cultural	**0.60 (0.50–0.72)**	**0.48 (0.35–0.65)**	**0.67 (0.55–0.82)**	**0.57 (0.41–0.79)**
Education	0.96 (0.79–1.19)	0.73 (0.52–1.01)	1.22 (0.95–1.57)	0.91 (0.63–1.32)
Administration	Reference	Reference	Reference	Reference
Sales	**0.72 (0.57–0.91)**	0.89 (0.63–1.26)	0.86 (0.66–1.12)	1.09 (0.76–1.57)
Construction	**0.83 (0.69–0.98)**	1.23 (0.97–1.56)	0.97 (0.79–1.19)	1.27 (0.97–1.66)
Other	**0.56 (0.45–0.68)**	0.93 (0.70–1.24)	**0.72 (0.57–0.91)**	1.06 (0.77–1.45)
Disease duration				
0–4 years	Reference	Reference	Reference	Reference
5–9 years	**1.20 (1.06–1.36)**	0.97 (0.81–1.17)	1.07 (0.94–1.23)	0.91 (0.75–1.11)
10–19 years	**1.79 (1.57–2.04)**	1.19 (0.98–1.44)	**1.41 (1.22–1.64)**	0.94 (0.76–1.15)
⩾20 years	**2.60 (2.05–3.29)**	**1.68 (1.24–2.27)**	**1.57 (1.20–2.04)**	1.04 (0.75–1.44)
Type of MS^c^				
Relapsing-remitting	Reference	Reference		
Primary progressive	**2.73 (2.11–3.53)**	**2.98 (2.27–3.90)**		
Comorbidity (categories)^d^				
0	Reference	Reference	Reference	Reference
1–2	**1.76 (1.48–2.09)**	1.26 (0.95–1.69)	**1.72 (1.43–2.06)**	1.27 (0.95–1.71)
3–4	**3.59 (2.97–4.34)**	**2.47 (1.83–3.33)**	**2.95 (2.41–3.62)**	**2.21 (1.62–3.00)**
⩾5	**6.72 (5.26–8.58)**	**5.10 (3.69–7.05)**	**5.42 (4.18–7.04)**	**4.19 (3.00–5.85)**

SA: sickness absence; DP: disability pension; MS: multiple sclerosis; SPDR: Swedish Prescribed Drug Register.

Bold values indicate statistical significance of *p* < 0.05.

a Adjusted for sex, age, educational level, country of birth, type of living area, type of occupation, disease duration and comorbidity.

b Individuals with missing variables added to lowest category, <0.75% of the cohort.

c Type of MS at diagnosis was not included in this mutually adjusted model, because of interrelatedness with other variables.

d Comorbidities are based on the SPDR and Swedish Cancer Register and categorized by a number of distinct comorbidity groups. The total number of comorbidities excludes MS.

Regarding the associations of types of occupations to the trajectories, Managers across all sectors (OR = 0.37, 95% CI = 0.26–0.52), Science & Technology (OR = 0.64, 95% CI = 0.50–0.82) and Economics, Social & Cultural (OR = 0.67, 95% CI = 0.55–0.82) were inversely associated with the *Moderate Increasing* group. Similarly, Managers (OR = 0.52, 95% CI = 0.30–0.89), Science & Technology (OR = 0.58, 95% CI = 0.39–0.88) and Economics, Social & Cultural (OR = 0.57, 95% CI = 0.41–0.79) were also inversely associated with the *High Increasing* group.

## Discussion

In this exploratory study, we investigated the types of occupations among working-aged PwMS and their annual levels of SA/DP net days, compared with matched references without MS from the general population. Insight into trajectories of SA/DP net days and associations to type of occupation was also obtained for PwMS. A steady increase of SA/DP net days over time was found for both PwMS and references in all types of occupations. Moreover, PwMS had higher levels of SA/DP compared with references in the same type of occupation, with a sevenfold higher level for Managers and fourfold for Healthcare, respectively. Among PwMS, Managers showed low levels of SA/DP, while Administration and Construction showed high levels of SA/DP. Three SA/DP trajectory groups were identified among PwMS: *Persistently Low, Moderate Increasing* and *High Increasing*, with most (55.2%) of PwMS included in the *Persistently Low* group, that is, they hardly had any SA/DP days at all during all follow-up years. Managers across all sectors and those working in Science & Technology, and Economics, Social & Cultural were more likely to belong to the *Persistently Low* group.

Our findings showed a steady increase in SA/DP net days over time among PwMS when stratified by sex, disease duration and type of occupation. Higher levels of SA/DP among PwMS compared with references are consistent with other studies. However, the studies differ in design, cohorts and study periods.^5,11,20–22^ In our study, we used a prevalence cohort which enabled us to present the level and course of SA/DP for both recently diagnosed PwMS and PwMS with long disease duration (i.e. ⩾20 years), therefore allowing exploration of the impact of the disease on SA/DP in the long term. Moreover, the impact of type of MS was also relevant in respect to mean annual SA/DP net days during the follow-up years, suggesting a greater loss of productivity if diagnosed with a more aggressive type of MS from the beginning (i.e. primary progressive MS type).

Regarding associations between type of occupation and SA/DP, there were lower levels of SA/DP among Managers, while Administration showed higher SA/DP levels. These lower SA/DP levels in Managers could be explained by higher percentage of men and shorter disease duration, as female sex and longer disease duration are known risk factors for SA/DP in PwMS.^21,23^ Similarly, high levels of SA/DP in Administration might be related to the larger proportion of PwMS with longer disease duration (⩾20 years). Moreover, office workers may have better opportunities for work adaptations, also linked to the possibility of switching to or staying in a less demanding administrative job when cognitive or physical limitations arise.^24^ Contrary to our results, a previous study with broad occupational categorisation (i.e. blue-collar and white-collar work) showed no association between type of work and future full-time DP,^22^ which could illustrate the need of categorisation on a finer scale concerning work and occupation.

With decreasing work capacity, SA transitions into DP. As we were interested in the level and course of both SA and DP, they were combined into one outcome variable. We identified three different trajectory groups (*Persistently Low, Moderate Increasing* and *High Increasing)*, while another study identified five trajectories of SA/DP among PwMS.^13^ However, the latter study used an incidence cohort and studied time around diagnosis, with this period having a significant impact on work capacity, compared with our inclusion of a prevalence cohort. The merit of using trajectories lays in the ability to identify different types of SA/DP trajectories over time instead of at a single point in time, providing a better representation of SA/DP courses throughout follow-up.

Our results also indicate the influence of educational level on the course of SA/DP, as lower education was associated with being in the *Moderate Increasing* and *High Increasing* groups. Although Education held the highest percentage of highly educated PwMS, no association between Education and level of SA/DP was found in the multinomial logistic regression analysis. The results were similar for Construction, who, in contrast, had a low percentage of highly educated PwMS. One might assume that other risk factors are of importance for the level of SA/DP, such as sex, disease duration or comorbidities. Although other studies showed that educational level had some association with SA/DP,^11,21^ Wiberg et al.^11^ found a stronger relation between office/manual work and current employment status than by educational level. In a smaller survey study, no association was found between educational level and later full-time DP, although education was considered important for SA/DP.^22^

The strength of this study lies in the prospective cohort design, a population-based study cohort with prevalent MS, including matched references, the use of high-quality register-based data, no loss to follow-up and information not biased by self-reported information. This enabled us to explore the long-term patterns of SA/DP days for PwMS, including those with long disease duration. However, limitations need to be addressed. First, the occupational categorisations do not allow for specific individual demands within each occupation, which would potentially impact remaining in work despite MS limitations. Moreover, we lack information on underemployed or ‘gig’ workers, underestimating the impact of work absenteeism in these groups. Second, we do not know if people remained in the same type of occupation throughout follow-up. Therefore, this study rather describes classification-based changes through time. Third, some limitations regarding register studies are present, such as unavailable clinical information, uncomplete data on workforce dropouts or information on shorter SA spells. However, this was also the situation for the references that we compared with and the shorter spells stand for a smaller number of SA days/year.

One can hypothesize that matching work demands and work capacity can aid PwMS to remain in paid work for more years. However, further research elucidating the associations between symptoms, the course of the different types of MS, treatments, life style, work adaptations, work demands and SA/DP and remaining in paid work is needed. For example, if new highly effective disease-modifying therapies could potentially modulate work capacity through time (e.g. reduce future absence from the workforce). Further analyses of PwMS in the *Persistently Low* group could provide valuable information on factors associated with remaining in paid work.

To conclude, this study shows an overall increasing number of SA/DP net days among PwMS in all types of occupations. Three SA/DP trajectory groups were identified among prevalent PwMS, with the majority belonging to the *Persistently Low* group. However, a significant part of PwMS belonged to the *Moderate Increasing* or *High Increasing* group. Several factors were associated with the *Moderate Increasing* or *High Increasing* groups, for example, low educational level and long disease duration. Managers across all sectors and those working in Science & Technology, and Economics, Social & Cultural were more likely to belong to the *Persistently Low* group. These findings suggest that type of occupation plays a role in the level and course of SA/DP.

## Supplemental Material

sj-docx-1-msj-10.1177_13524585211048759 – Supplemental material for Trajectories of sickness absence and disability pension days among people with multiple sclerosis by type of occupation Click here for additional data file. Supplemental material, sj-docx-1-msj-10.1177_13524585211048759 for Trajectories of sickness absence and disability pension days among people with multiple sclerosis by type of occupation by Astrid R. Bosma, Chantelle Murley, Jenny Aspling, Jan Hillert, Frederieke G. Schaafsma, Johannes R. Anema, Cécile R.L. Boot, Kristina Alexanderson, Alejandra Machado and Emilie Friberg in Multiple Sclerosis Journal
