# Supplementary material for: Trajectories of sickness absence and disability pension days among people with multiple sclerosis by type of occupation
Source: Mult Scler. 2021 Oct 6;28(9):1402–13. doi: 10.1177/13524585211048759 (PMC9260482; doi:10.1177/13524585211048759)
Supplement: sj-docx-1-msj-10.1177_13524585211048759 – Supplemental material for Trajectories of sickness absence and disability pension days among people with multiple sclerosis by type of occupation [file sj-docx-1-msj-10.1177_13524585211048759.docx]

Trajectories of sickness absence and disability pension days among people with multiple sclerosis by type of occupation

Astrid R. Bosma, Chantelle Murley, Jenny Aspling, Jan Hillert, Frederieke G. Schaafsma, Johannes R. Anema, Cécile R.L. Boot, Kristina Alexanderson, Alejandra Machado and Emilie Friberg

Supplementary file


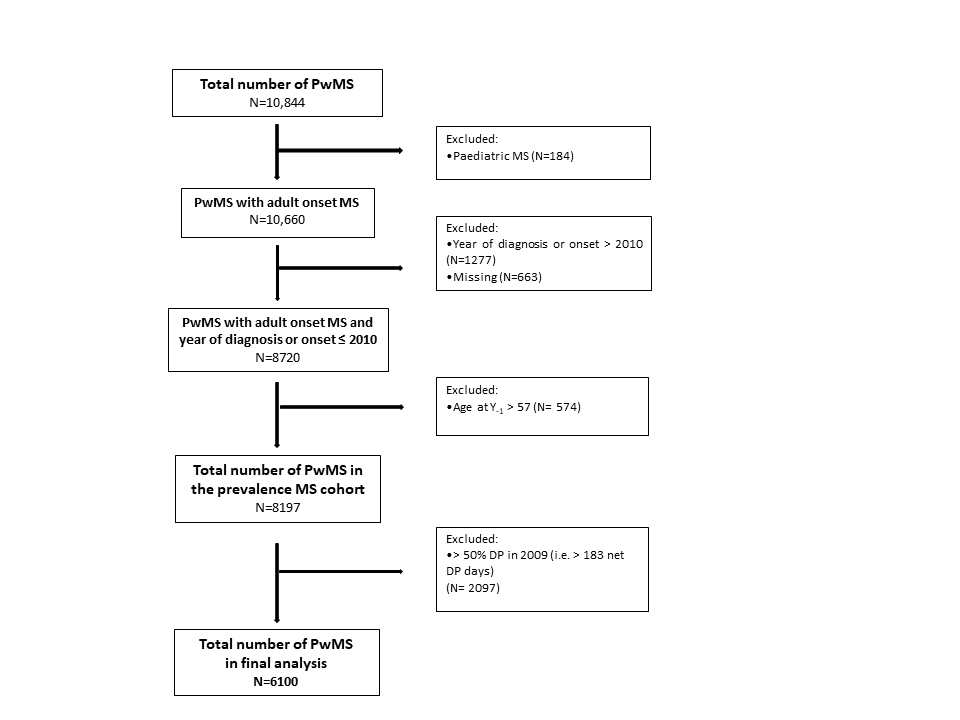


**Supplementary Figure 1.** Flowchart of creation of the prevalence MS cohort and group of people with MS (PwMS) included in the analysis.

**Supplementary Table 1.** Baseline characteristics in 2009 of people with MS (PwMS) and references - including people on >50% disability pension (DP).

|  | **PwMS**  n= 8197 (%) | **References**  n= 40,985 (%) |
| --- | --- | --- |
| **Sex** |  |  |
| Women | 5902 (72.0) | 29510 (72.0) |
| Men | 2295 (28.0) | 11475 (28.0) |
|  |  |  |
| **Age (years)** |  |  |
| 19-24 | 264 (3.2) | 1320 (3.2) |
| 25-34 | 1443 (17.6) | 7215 (17.6) |
| 35-44 | 2728 (33.3) | 13640 (33.3) |
| 45-54 | 2950 (36.0) | 14750 (36.0) |
| 55-57 | 812 (9.9) | 4060 (9.9) |
| Mean age (SD) | 42.6 (9.2) | 42.6 (9.2) |
|  |  |  |
| **Educational level** |  |  |
| Low: Compulsory school ≤9 years)^a^ | 879 (10.7) | 5360 (13.1) |
| Medium: Upper secondary school 10-12 years | 4019 (49.0) | 19160 (46.7) |
| High: Higher education >12 years | 3299 (40.3) | 16465 (40.2) |
|  |  |  |
| **Married or in civil partnership** |  |  |
| No | 4547 (55.5) | 21709 (53.0) |
| Yes | 3650 (44.5) | 19276 (47.0) |
|  |  |  |
| **Living with children aged <18 years** |  |  |
| No | 4756 (58.0) | 21136 (51.6) |
| Yes | 3441 (42.0) | 19849 (48.4) |
|  |  |  |
| **Country of birth** |  |  |
| Sweden | 7380 (90.0) | 33417 (81.5) |
| Outside of Sweden | 817 (10.0) | 7568 (18.5) |
|  |  |  |
| **Type of living area** |  |  |
| Larger cities | 3246 (39.6) | 16230 (39.6) |
| Medium-sized municipalities | 2832 (34.5) | 14160 (34.5) |
| Smaller municipalities | 2119 (25.9) | 10595 (25.9) |
|  |  |  |
| **Type of occupation** |  |  |
| Managers across all sectors | 237 (2.9) | 1808 (4.4) |
| Science & Technology | 567 (6.9) | 2663 (6.5) |
| Healthcare | 1538 (18.7) | 8607 (21.0) |
| Economics, Social & Cultural | 898 (11.0) | 3988 (9.7) |
| Education | 534 (6.5) | 3117 (7.6) |
| Administration | 1337 (16.3) | 5798 (14.1) |
| Sales | 406 (5.0) | 2116 (5.2) |
| Construction | 1105 (13.5) | 6624 (16.2) |
| Other | 1575 (19.2) | 6264 (15.3) |
|  |  |  |
| **Employment status**^b^ |  |  |
| In paid work | 5439 (66.4) | 32848 (80.1) |
| Not in paid work | 2758 (33.6) | 8137 (19.9) |
|  |  |  |
| **Receiving student allowances** |  |  |
| No | 7858 (95.9) | 38813 (94.7) |
| Yes | 339 (4.1) | 2172 (5.3) |
|  |  |  |
| **Receiving parental leave benefits** |  |  |
| No | 7220 (88.1) | 34998 (85.4) |
| Yes | 977 (11.9) | 5987 (14.6) |
| **SA/DP net days** |  |  |
| Mean SA/DP net days 2009 | 139 | 33 |
|  |  |  |
| **Comorbidity (categories)**^c^ |  |  |
| 0 | 848 (10.3) | 13002 (31.7) |
| 1-2 | 4073 (49.7) | 19231 (46.9) |
| 3-4 | 2163 (26.4) | 6104 (14.9) |
| ≥ 5 | 1113 (13.6) | 2648 (6.5) |
|  |  |  |
| **Disease duration** |  |  |
| 0-4 years | 2770 (33.8) | - |
| 5-9 years | 2363 (28.8) | - |
| 10-19 years | 2330 (28.4) | - |
| ≥ 20 years | 734 (9.0) | - |
| Mean disease duration in years (SD) | 8.7 (7.2) | - |
|  |  |  |
| **Type of MS** |  |  |
| Relapsing-remitting | 7422 (90.5) | - |
| Primary progressive | 775 (9.5) | - |

A comparable reference group from the general population, without MS, were matched on sex, age, type of living area and county. For each MS individual, up to five references were included (ratio 1:5).

Variables are described by frequencies and percentages or mean and standard deviation (SD).

MS-related variables were described solely for all PwMS.

^a^ Individuals with missing variables added to lowest category, <0.75% of the cohort.

^b^ Individuals unemployed, on parental leave or students (receiving student allowances) were included as “not in paid work”.

^c^ Comorbidities are based on the SPDR and Swedish Cancer Register and categorized by a number of distinct comorbidity groups. The total number of comorbidities excludes MS.

**Supplementary Table 2.** Description of the types of occupations

| **Type of occupation** | **Description** | **Examples** |
| --- | --- | --- |
| Managers across all sectors | Managers and senior officials in various work sectors, including e.g., finance, healthcare, politics and education | Chief Executive Officer, Hospital director, University rector |
| Science & Technology | Occupations in science, technology and IT that require higher educational (university) qualifications | Architect, Civil engineer, Data analyst |
| Healthcare | Occupations in health care, medical care, and laboratory medicine, including care professions | Home caregiver, Laboratory assistant, Psychologist, Surgeon |
| Economics, Social & Cultural | Occupations in economics, law, culture and social work, that require higher educational (university) qualifications | Journalist, Lawyer, Social worker, Stockbroker |
| Education | Occupations in education at different levels and various specialist competencies | University lecturer. Middle school teacher, Handcraft teacher |
| Administration | Occupations in office and secretary work, and customer service professions | Library clerk, Payroll administrator, Secretary |
| Sales | Occupations in sales, including retail | Car salesperson, Cashier, Telemarketer |
| Construction | Occupations in construction, manufacturing transport, cleaning, and hospitality | Baker, Crane driver, Electrician |
| Other | Unspecified and other occupations, like military and agriculture | Dairy farmer, Soldier |

Note: The type of occupation was based on the 1996 SSYK codes although renamed and regrouped into broader categories based on the 2012 SSYK codes - consisting of 10 occupational areas. In this study, however, we included the “military sector” in the “other” group as it contained very few individuals, therefor 9 types of occupations in our study.

**a**


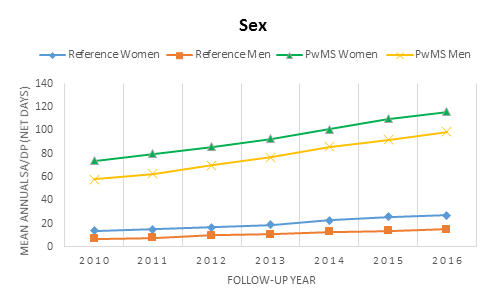


**b**


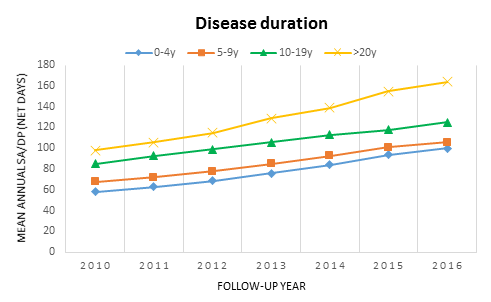


**Supplementary Figure 2 a-b**. Mean annual sickness absence (SA)/disability pension (DP) (net days) for both people with MS (PwMS) and references stratified by sex (a) and for PwMS stratified by disease duration (b).

**Supplementary Table 3.** Mean annual sickness absence (SA)/disability pension (DP) net days among people with MS (PwMS) by type of MS and for each follow-up year.

| **Year**  **Types of MS** | **2010** | **2011** | **2012** | **2013** | **2014** | **2015** | **2016** |
| --- | --- | --- | --- | --- | --- | --- | --- |
| Relapsing-remitting | 68 (101.6) | 72.9 (108.1) | 78.7 (112.7) | 86.3 (118.2) | 93.9 (124.5) | 102 (128.7) | 108.1 (133.5) |
| Primary progressive | 107.2 (125.7) | 125.9 (136.5) | 142.2 (140.4) | 149.7 (142.6) | 163.6 (146.4) | 180.5 (146.9) | 190.3 (151) |

Note: The type of MS classification is based on the presumption for MS type at diagnosis. Thus, the relapsing-remitting group at diagnosis also includes those that convert to secondary progressive before 2010 or during follow-up years. The primary progressive MS type also includes the progressive relapsing type.

Annual SA/DP values are described as mean and standard deviation (SD).

Comparisons between groups were statistically significant for all follow-up years (p<0.001).

**Supplementary Table 4.** Distribution among people with MS (PwMS, n=6100) and references (n=38,641) who obtain full disability pension (≥75% of DP) for each follow-up year.

|  | **2010** | **2011** | **2012** | **2013** | **2014** | **2015** | **2016** |
| --- | --- | --- | --- | --- | --- | --- | --- |
| **PwMS,** n (%) | 87 (1.4) | 165 (2.7) | 271 (4.4) | 352 (5.8) | 468 (7.7) | 585 (9.6) | 672 (11.0) |
| **References,** n (%) | 36 (0.1) | 74 (0.2) | 115 (0.3) | 182 (0.5) | 261 (0.7) | 334 (0.9) | 413 (1.1) |

**Supplementary Table 5. Baseline characteristics of people with MS (PwMS) in the trajectory groups**

|  | Persistently Low  n= 3371 (%) | Moderate Increasing  n= 1949 (%) | High Increasing  n=780 (%) |
| --- | --- | --- | --- |
| **Sex** |  |  |  |
| Women | 2262 (67.1) | 1536 (78.8) | 557 (71.4) |
| Men | 1109 (32.9) | 413 (21.2) | 223 (28.6) |
|  |  |  |  |
| **Age (years)** |  |  |  |
| 19-24 | 208 (6.2) | 42 (2.1) | 7 (0.9) |
| 25-34 | 913 (27.1) | 311 (16.0) | 95 (12.2) |
| 35-44 | 1278 (37.9) | 676 (34.7) | 244 (31.3) |
| 45-54 | 804 (23.8) | 736 (37.8) | 355 (45.5) |
| 55-57 | 168 (5.0) | 184 (9.4) | 79 (10.1) |
|  |  |  |  |
| **Educational level** |  |  |  |
| Low: Compulsory school ≤9 years)^a^ | 202 (6.0) | 164 (8.4) | 99 (12.7) |
| Medium: Upper secondary school 10-12 years | 1415 (42.0) | 956 (49.1) | 423 (54.2) |
| High: Higher education >12 years | 1754 (52.0) | 829 (42.5) | 258 (33.1) |
|  |  |  |  |
| **Married or in civil partnership** |  |  |  |
| NoN | 1899 (6.3) | 1024 (52.5) | 435 (55.8) |
| Yes | 1472 (43.7) | 925 (47.5) | 345 (44.2) |
|  |  |  |  |
| **Living with children aged <18 years** |  |  |  |
| No | 1726 (51.2) | 1038 (53.3) | 466 (59.7) |
| Yes | 1645 (48.8) | 911 (46.7) | 314 (40.3) |
|  |  |  |  |
| **Country of birth** |  |  |  |
| Sweden | 3038 (90.1) | 1794 (92.0) | 692 (88.7) |
| Outside of Sweden | 333 (9.9) | 155 (8.0) | 88 (11.3) |
|  |  |  |  |
| **Type of living area** |  |  |  |
| Larger cities | 1542 (45.7) | 711 (36.5) | 268 (34.4) |
| Medium-sized municipalities | 1122 (33.3) | 683 (35.0) | 285 (36.5) |
| Smaller municipalities | 707 (21.0) | 555 (28.5) | 227 (29.1) |
|  |  |  |  |
| **Type of occupation** |  |  |  |
| Managers across all sectors | 159 (4.7) | 39 (2.0) | 18 (2.3) |
| Science & Technology | 367 (10.9) | 125 (6.4) | 34 (4.4) |
| Healthcare | 570 (16.9) | 447 (22.9) | 160 (20.5) |
| Economics, Social & Cultural | 486 (14.4) | 249 (12.8) | 62 (7.9) |
| Education | 226 (6.7) | 180 (9.2) | 52 (6.7) |
| Administration | 534 (15.9) | 408 (20.9) | 166 (21.3) |
| Sales | 203 (6.0) | 108 (5.5) | 49 (6.3) |
| Construction | 479 (14.2) | 264 (13.6) | 161 (20.6) |
| Other | 347 (10.3) | 129 (6.7) | 78 (10.0) |
|  |  |  |  |
| **Disease duration** |  |  |  |
| 0-4 years | 1552 (46.0) | 679 (34.8) | 306 (39.2) |
| 5-9 years | 1033 (30.7) | 573 (29.4) | 214 (27.4) |
| 10-19 years | 661 (19.6) | 553 (28.4) | 198 (25.4) |
| ≥ 20 years | 125 (3.7) | 144 (7.4) | 62 (8.0) |
|  |  |  |  |
| **Type of MS** |  |  |  |
| Relapsing-remitting | 3160 (93.7) | 1794 (92.0) | 681 (87.3) |
| Primary progressive | 211 (6.3) | 155 (8.0) | 99 (12.7) |

Variables are described by frequencies and percentages or mean and standard deviation (SD).

^a^ Individuals with missing variables added to lowest category, <0.75% of the cohort.
